# The anti-leukemic activity of sodium dichloroacetate in p53^mutated/null^ cells is mediated by a p53-independent ILF3/p21 pathway

**DOI:** 10.18632/oncotarget.2960

**Published:** 2014-12-10

**Authors:** Chiara Agnoletto, Laura Brunelli, Elisabetta Melloni, Roberta Pastorelli, Fabio Casciano, Erika Rimondi, Gian Matteo Rigolin, Antonio Cuneo, Paola Secchiero, Giorgio Zauli

**Affiliations:** ^1^ Department of Morphology, Surgery and Experimental Medicine and LTTA Centre, University of Ferrara, Ferrara, Italy; ^2^ Institute of Pharmacological Researches, IRCCS “Mario Negri”, Milano, Italy; ^3^ Department of Life Science, University of Trieste, Trieste, Italy; ^4^ Department of Medical Sciences, University of Ferrara-Arcispedale S. Anna, Ferrara, Italy; ^5^ Institute for Maternal and Child Health, IRCCS “Burlo Garofolo”, Trieste, Italy

**Keywords:** Sodium dichloroacetate, B-CLL, p21, proteomics, cytotoxicity

## Abstract

B-chronic lymphocytic leukemia (B-CLL) patients harboring p53 mutations are invariably refractory to therapies based on purine analogues and have limited treatment options and poor survival. Having recently demonstrated that the mitochondria-targeting small molecule sodium dichloroacetate (DCA) exhibits anti-leukemic activity in p53^wild-type^ B-CLL cells, the aim of this study was to evaluate the effect of DCA in p53^mutated^ B-CLL cells and in p53^mutated/null^ leukemic cell lines. DCA exhibited comparable cytotoxicity in p53^wild-type^ and p53^mutated^ B-CLL patient cell cultures, as well as in p53^mutated^ B leukemic cell lines (MAVER, MEC-1, MEC-2). At the molecular level, DCA promoted the transcriptional induction of p21 in all leukemic cell types investigated, including p53^null^ HL-60. By using a proteomic approach, we demonstrated that DCA up-regulated the ILF3 transcription factor, which is a known regulator of p21 expression. The role of the ILF3/p21 axis in mediating the DCA anti-leukemic activity was underscored by knocking-down experiments. Indeed, transfection with ILF3 and p21 siRNAs significantly decreased both the DCA-induced p21 expression and the DCA-mediated cytotoxicity. Taken together, our results emphasize that DCA is a small molecule that merits further evaluation as a therapeutic agent also for p53^mutated^ leukemic cells, by acting through the induction of a p53-independent pathway.

## INTRODUCTION

Emerging data indicate that cancer associated oxidative stress, resulting from the accumulation of reactive oxygen species and leading to genetic instability and drug resistance [1,2], might be involved in the pathogenesis of B-chronic lymphocytic leukemia (B-CLL) [3]. A recent study has demonstrated the prevalence of oxidative stress in B-CLL patients, adaptation to permanent intrinsic oxidative stress and mitochondrial biogenesis [4]. Thus, the mitochondria might be primary targets of cancer therapeutics instead of simple bystanders during cancer development. In this context, there is an increasing interest for the therapeutic potentiality of the mitochondria-targeting dichloroacetate (DCA) against malignant cells. Indeed, preclinical studies confirm the relatively low toxicity of DCA and document its efficacy against numerous epithelial cancers (such as non-small cell lung, endometrial, prostate, breast and colorectal cancer), glioblastoma, as well as multiple myeloma [5-14]. Recently, we have demonstrated that DCA is cytotoxic also in p53^wild-type^ primary B-CLL patient cells and in p53^wild-type^ B leukemic cell lines [15], when used in the same range of concentrations previously employed in *in vitro* studies performed in solid tumors and multiple myeloma cells [10-12,14]. Moreover, we found that in p53^wild-type^ B leukemic cells DCA activates p53 and potently synergizes with Nutlin-3, a non-genotoxic activator of the p53 pathway [15].

Although these findings were promising, a major unresolved clinical problem of B-CLL is represented by the lack of effective treatments for B-CLL patients harboring TP53 mutations [16]. In this respect, although TP53 mutations in naïve B-CLL were usually considered a rare (<5%) event [16-17], a recent study performed using the next generation sequencing technology demonstrated that very small TP53 mutated subclones are present in 9% (28/309) of newly diagnosed B-CLL patients [18], a percentage significantly higher than previously reported by the Sanger technology. Of note, patients harboring small TP53 mutated subclones showed the same clinical phenotype and poor survival as patients carrying clonal TP53 lesions [18]. In addition, the percentage of TP53 mutations dramatically increases up to >30% after relapsed chemotherapy [19].

On these bases, the aim of the present study was to evaluate the potential therapeutic activity of DCA in p53^mutated^ B leukemic cells. For this purpose, DCA cytotoxicity was evaluated on primary p53^mutated^ B-CLL patient cells in comparison with p53^wild-type^ B-CLL patient cells as well as on a panel of p53^mutated^ B leukemic cell lines (MAVER, MEC-1, MEC-2). Finally, in order to dissect the p53-independent molecular mechanisms of DCA cytotoxicity, a set of experiments was performed using the p53^null^ HL-60 leukemic cell line.

## RESULTS

### DCA promotes comparable cytotoxicity in p53^wild-type^ and p53^mutated^ B-CLL patient cells

Since B-CLL patients characterized by p53 dysfunction have limited treatment options and poor overall survival [16,18,19], in the first set of experiments we comparatively evaluated the *in vitro* effect of DCA assessed on B-CLL patient cells characterized by either p53 wild-type or harboring TP53 mutations (Table 1). For this purpose, upon validation of a TP53 next generation sequencing screening (performed on a total of 80 B-CLL patients), we selected 5 patients with p53 wild-type and 5 patients characterized by mutations potentially affecting p53 functionality, as predicted by web mutation pathogenicity prediction tools and protein structural bioinformatic analysis (Table 1 and Supplementary Figure 1). B-CLL cell cultures were exposed *in vitro* to DCA in a range of concentrations (1-30 mM) previously used by other authors in *in vitro* solid tumor models [10-12,14], and in our recent study performed in primary p53^wild-type^ B-CLL cells [15]. As documented by the IC_50_ (50% inhibition concentration) values, *in vitro* treatment with DCA induced a significant and progressive reduction of cell viability, with respect to the untreated cultures assessed at the same time points (24 and 48 hours), in all the primary B-CLL patient cell cultures, irrespectively of the p53 status (Table 2).

**Table 1:** Clinical and laboratory characteristics of the B-CLL patients

B-CLL Pt.^#^	Sex	Age	Rai stage	Therapy	%CD38^+^ cells	%ZAP-70+	Cytogenetic Abnormalities *	IgVH status	TP53 analysis (NGS)
1	M	73	0	none	10.3	10.2	del13q	mut	unmut
2	F	78	0	F	6.3	1.8	del11q/del13q omoz.	unmut	unmut
3	M	63	0	FCR	0	24.3	del13q	unmut	unmut
4	M	54	II	FCR	1.2	60	normal	unmut	unmut
5	M	77	IV	Chl	15.4	72.1	tris 12	unmut	unmut
6	M	55	0	FCR	6.7	18.9	tris 12	unmut	NM_000546:exon5:c.470T>A:p.V157D
7	M	71	IV	FCR	6.5	na	del13q/del17p	mut	NM_000546:exon8:c.817C>T:p.R273C NM_000546:exon7:c.701A>G:p.Y234C
8	M	68	IV	R-Benda	16.6	8.6	del11q/del13q/del17p	unmut	NM_000546:exon7:c.770T>C:p.L257P
9	M	79	II	Chl	14.3	1.9	del13q/del17p	unmut	NM_000546:exon6:c.376-2A>G: p.Y126Tfs*44
10	M	75	0	Chl	3.6	41.1	del13q/del17p	na	NM_000546:exon8:c.832C>T:p.P278S

Pt., patient; NGS, next generation sequencing; mut, mutated; unmut, unmutated; na, not available; F, Fludarabine; FCR, Fludarabine-Cyclophosphamide-Rituximab; R-Benda, Rituximab-Bendamustine; Chl, Chlorambucil.

* Cytogenetic abnormalities were evaluated by fluorescence in situ hybridization (FISH) analysis.

**Table 2:** IC_50_ for DCA in leukemic cells

Leukemic cells	TP53 status	IC_50_ DCA (mM)	
		24 hours	48 hours
B-CLL Pt.#1	wild-type	20.6	14.7
B-CLL Pt.#2	wild-type	24.8	12.7
B-CLL Pt.#3	wild-type	28.5	9.7
B-CLL Pt.#4	wild-type	23.1	16.5
B-CLL Pt.#5	wild-type	36.2	<1
B-CLL Pt.#6	mutated	41.9	20.4
B-CLL Pt.#7	mutated	32.1	15.0
B-CLL Pt.#8	mutated	5.0	<1
B-CLL Pt.#9	mutated	17.0	15.6
B-CLL Pt.#10	mutated	28.1	14.8
MAVER	mutated	27.9	14.5
MEC-1	mutated	16.2	10.4
MEC-2	mutated	33.7	22.7
HL-60	null	43.0	24.4

**Figure 1:**
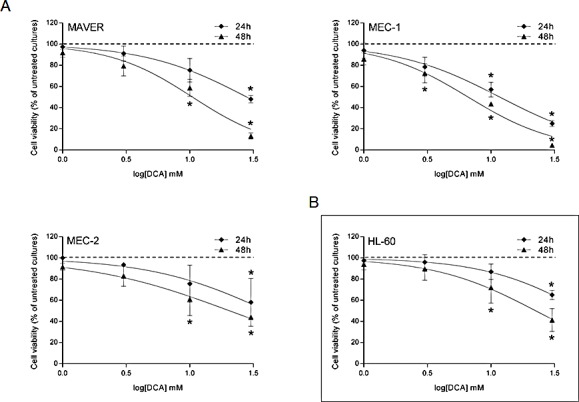
Cytotoxicity induced by DCA in p53^mutated/null^ leukemic cell lines The p53^mutated^ B leukemic cell lines MAVER, MEC-1 and MEC-2 (A), as well as the p53^null^ HL-60 cells (B), were exposed to serial doses of DCA (range 1-30 mM) before analysis of cell viability at both 24 and 48 hours of treatment. Dose response curves were derived by calculating the cell viability as percentage with respect to the untreated cultures (set to 100%). Data are means±SD of at least three independent experiments, each performed in duplicate (Mann-Whitney rank-sum test; asterisks, p<0.05).

**Figure 2:**
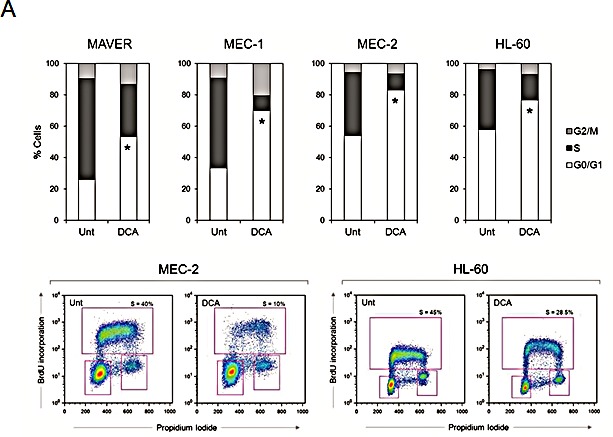

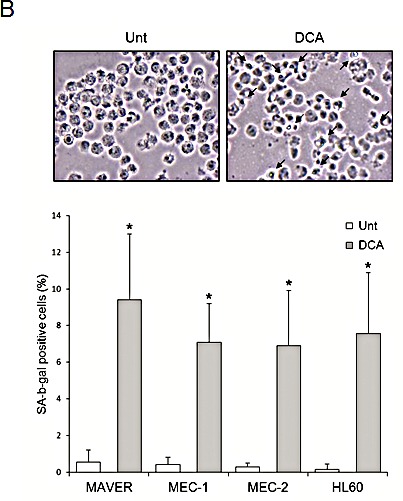

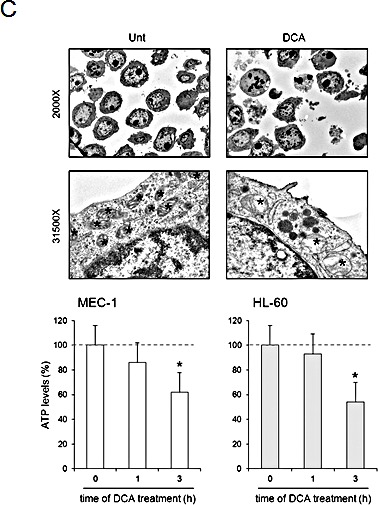

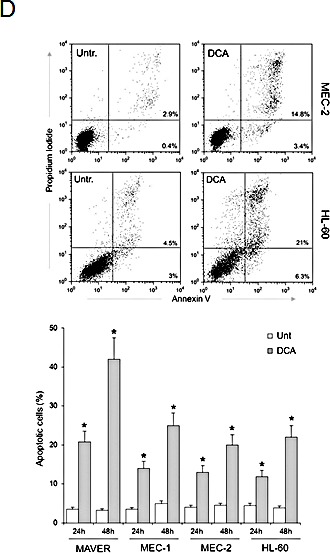
Effects of DCA treatment on p53^mutated/null^ leukemic cell lines The p53^mutated^ B leukemic cell lines MAVER, MEC-1 and MEC-2, as well as the p53^null^ HL-60 cells, were exposed to DCA (30 mM). In A, cell distribution in the different phases of the cell cycle was calculated from the flow cytometry dot plots after BrdU/PI staining and expressed as percentage of the total population. In the upper panel, data are reported as the means of results from at least three independent experiments (Mann-Whitney rank-sum test; asterisks, p<0.05). In the lower panel, representative cell cycle profiles of cultures, either left untreated or treated with DCA, analyzed by flow cytometry are shown. For each cytofluorimetric analysis, the rectangles represent the cells in G0/G1, S, G2/M phases of the cell cycle. In B, cell senescence was visualized by SA-β-gal staining. Representative fields observed under light microscope are shown (arrows, senescent cells); cells with positive staining for SA-β-gal were quantified by counting a total of 1000 cells for each culture condition. Data are expressed as means±SD of six different fields in three independent experiments (Mann-Whitney rank-sum test; asterisks, p<0.05). In C, assessment of mitochondrial alterations was carried out by morphological and functional analyses. Transmission electron microscopy images of cytosolic regions show mitochondria (asterisks; original magnification 31,500X) with either normal morphology or cristae remodeling, in untreated or DCA treated cultures, respectively. Representative fields of three independent experiments are shown. In the lower panel, cellular ATP levels were quantified in cell lysates at the indicated time points. Data are reported as percentage with respect to the untreated cultures (set to 100%) and are means±SD of three independent experiments, each performed in duplicate (Mann-Whitney rank-sum test; asterisks, p<0.05). In D, the percentage of apoptotic cells was determined by flow cytometry after Annexin V/PI staining. Representative experiments are shown. In the graph, data are reported as the means±SD of results from at least three independent experiments (Mann-Whitney rank-sum test; asterisks, p<0.05).

Although we have previously shown that DCA activates the p53 pathway in p53^wild-type^ B leukemic cells [15], the current set of data suggested that DCA can promote cytotoxicity also independently of functional p53. Thus, to investigate the molecular mechanisms underlining DCA cytotoxicity in leukemic cells with dysfunctional p53, we selected three p53^mutated^ B leukemic cell lines (MAVER, MEC-1, MEC-2), which exhibited a dose- and time-dependent cytotoxic response to DCA (Figure 1A) with IC_50_ values comparable to those assessed for primary p53^wild-type^ and p53^mutated^ B-CLL cells (Table 2). The ability of DCA to promote p53-independent cytotoxicity in leukemic cells was further supported by experiments performed on the p53^null^ HL-60 cell line (Figure 1B). The cytotoxicity induced by DCA in p53^mutated/null^ leukemic cell lines was the result of (i) a cytostatic effect with the accumulation in G1 phase of the cell cycle (Figure 2A), (ii) the promotion of cellular senescence (Figure 2B), (iii) the induction of mitochondrial damage (Figure 2C), coupled to (iv) apoptosis (Figure 2D).

### DCA induces the transcription of p21 in both p53^wild-type^ and p53^mutated/null^ leukemic cells

**Figure 3:**
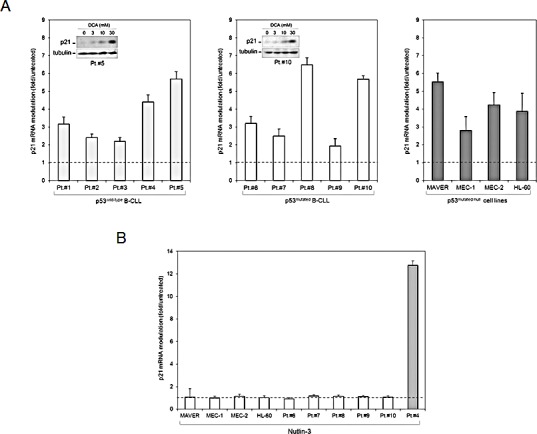
Induction of p21 by DCA in p53^mutated/null^ leukemic cells Levels of p21 were analyzed by quantitative RT-PCR in the p53^wilde-type^ and p53^mutated^ B-CLL patient samples, as well as in the p53^mutated^ B leukemic cell lines (MAVER, MEC-1 and MEC-2) and in the p53^null^ HL-60 cells. In A, levels of p21 modulation upon 24 hours of treatment with DCA (30 mM) are shown. Representative western blot results documenting induction of p21 protein by DCA in 2 B-CLL patient samples are shown in the insets. In B, the same cell cultures were exposed for 24 hours to Nutlin-3 (10 μM) before measurement of p21 levels. In parallel, as a positive control, p21 induction by Nutlin-3 was assessed in a p53^wild-type^ B-CLL sample. In A and B, mRNA levels are expressed as fold of modulation with respect to the control untreated cultures set at 1. Results are reported as means±SD of at least three independent experiments, performed in duplicate, carried out on each leukemic sample.

In the next set of experiments, we have investigated the mRNA levels of p21, whose *in vitro* induction represents a positive prognostic marker of response to therapy [20]. Surprisingly, DCA was equally effective in increasing the transcription levels of p21 in both primary p53^wild-type^ and p53^mutated^ B-CLL leukemic cells (Figure 3A). The induction of p21 was confirmed at the protein level by Western blot analysis. Moreover, DCA significantly increased the steady-state mRNA levels in all the p53^mutated^ B cell lines (MAVER, MEC-1 and MEC-2) as well as in p53^null^ HL-60 (Figure 3A), thus confirming the ability of DCA to induce p21 expression independently of the presence of p53. In parallel, a confirmation of the lack of functional p53 transcriptional activity in both p53^mutated^ primary B-CLL cells and p53^mutated^ MAVER, MEC-1 and MEC-2 cell lines as well as in p53^null^ HL-60 was provided by the use of Nutlin-3, a small molecule activator of p53 [21]. Indeed, while Nutlin-3 (10 μM) potently induced p21 transcription in primary p53^wild-type^ B-CLL cells, it did not promote any modulation of p21 mRNA over the baseline in both primary p53^mutated^ B-CLL patient samples and p53^mutated/null^ leukemic cell lines (Figure 3B), thus confirming the p53-independent activation of p21 by DCA in these cell models.

### Role of ILF3-p21 axis in DCA induced cytotoxicity

**Figure 4:**
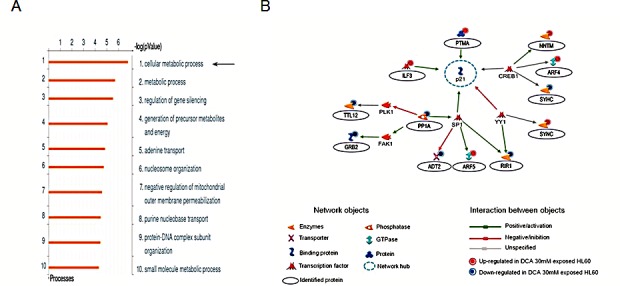

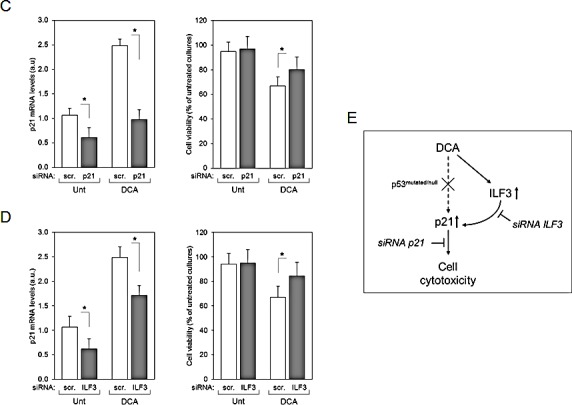
Role of p21 and ILF3 pathway in mediating the anti-leukemic activity of DCA in p53^null^ leukemic cells In A-B, proteomic profiling in HL-60 cells upon DCA treatment revealed significant different (at least 3 folds) levels for 17 proteins in DCA treated cultures compared to the untreated cells (Supplementary Table 2). In A, the top ten GO processes, prioritized in according to p-value, in response to DCA treatment is shown. In B, the protein network generated by the shortest path algorithm using the list of the differently expressed proteins (more than 3 fold induction/reduction) is reported. Nodes represent proteins and the different shapes of the nodes represent the functional class of the proteins. Circles evidence protein identified in our proteomic analysis. Lines connecting the nodes indicate indirect interactions (activation, induction, modification) or direct binding; the arrowheads indicate the direction of the interaction. Blue broken line circles highlight network hubs as indicated by network statistics. In C-D, HL-60 cells were transfected with either control scrambled (scr) siRNA, or p21 siRNA (C) or ILF3 siRNA (D) before treatment with DCA (30 mM). After transfection, levels of p21 mRNA were analyzed by quantitative RT-PCR (C-D). Data are expressed as arbitrary units (a.u.). In parallel transfected cultures were analyzed for cell viability upon exposure to DCA; results are expressed as percentage of viable cells with respect to the control cultures (set to 100%). Data are reported as means±SD of results from three independent experiments, each performed in duplicate (Mann-Whitney rank-sum test; asterisk, p<0.05). In E, a schematic representation of the potential p53-independent pathway mediating the anti-leukemic activity of DCA is shown.

To gain insight into the p53-independent induction of p21 by DCA, we adopted a proteomic approach using the HL-60 p53^null^ leukemic model in order to rule out any potential interference by mutated p53 protein. For this purpose, we used a 1-DE gel approach for protein pre-fractionation integrated into a typical LC-MS/MS workflow for protein identification, performed as previously described [22]. A label-free approach (spectral counting) was then used for relative protein abundance quantification. Overall, our proteomic analysis identified 727 proteins in the total lysate of untreated and DCA treated HL-60 cells. According to statistical tests, HL-60 exposed to DCA for 24 hours exhibited a significant difference (at least 3-fold) in the abundance of 17 proteins compared to the untreated counterpart, with 10 up-regulated proteins and 7 down-regulated proteins (Supplementary Tables 1 and 2). Network analysis, using MetaCore bioinformatic tools of the 17 significantly deregulated proteins, revealed the involvement of *Cellular metabolic processes* as the main GO processes altered by DCA treatment (Figure 4A). In addition, when MetaCore software was used to map the shortest paths of interactions among the differentially expressed proteins, 12 out of 17 proteins were brought together with p21 as the hub of the identified network (Figure 4B). Interestingly p21 was directly connected with interleukin enhancer-binding factor 3 (ILF3, also known as NFAT-90).

On the base of the proteomic results, we next tested the potential role of p21 in mediating the anti-leukemic activity of DCA using again the p53^null^ HL-60 cells as model system, by utilizing siRNAs to attenuate p21 expression. As shown in Figure 4C, once confirmed the effective knock-down of p21 expression through quantitative RT-PCR both at baseline and after DCA treatment, we observed a significant (p<0.05) reduction in DCA-induced cytotoxicity in p21 siRNA transfected cells with respect to cells transfected with a scrambled control siRNA. Concurrently, p21 silencing reversed the cell cycle arrest, as documented by the minor extent in cell blockage in S phase upon DCA treatment (data not shown). This set of data confirmed that p21 plays a central role in mediating cytotoxicity of DCA not only in p53^wild-type^ B leukemic cells [15] but also in p53^deficient^ leukemic cells.

Subsequently, in order to disclose the mechanism of p21 regulation by DCA, we attenuate ILF3 expression. As shown in Figure 4D, the knock-down of ILF3 expression by siRNA transfection determined the concomitant reduction in p21 mRNA levels both in untreated culture and upon DCA exposure, consistently with the documented role of ILF3 as a regulator of p21 at transcriptional and post-transcriptional levels [23,24] as summarized in Supplementary Figure 2. The involvement of ILF3 in DCA anti-leukemic signaling pathway, in absence of functional p53 pathway, was revealed by the effect of its down-modulation in attenuating DCA cytotoxicity, similarly to the effect resulted by p21 gene silencing (Figure 4D-E). On the other hand, knock-down of ILF3 expression in p53^wilde-type^ cells did not determine significant modulatory effects on p21 (both in untreated culture and upon DCA exposure; Supplementary Figure 3). Although this is not a direct evidence of the role of p53 in p21 regulation, nevertheless it suggests a prominent role of p53-pathway in the p21 induction by DCA in p53^wild-type^ cells.

## DISCUSSION

Agents directed against the unique metabolic features of cancer cells might represent an opportunity to improve current treatments, in particular with the aim of overcoming drug resistance in leukemic cells. In this context, we have recently demonstrated that the mitochondria-targeting small molecule DCA promoted cytotoxicity in p53^wild-type^ primary B-CLL cells and B leukemic cell lines at concentrations which marginally affected primary normal peripheral blood mononuclear cells [15]. However, p53 mutations are known to interfere with the normal response of human cells to oxidative stress. The failure of p53 mutant-expressing cells to restore a reducing oxidative environment is often accompanied by increased survival [25], which represents an aspect of the gain of function of the pro-oncogenic activity of mutated TP53. Thus, it remained to be established whether DCA was active also on p53^mutated^ leukemic cells.

In this study, we have demonstrated for the first time that DCA exerts cytotoxic activity in both primary p53^mutated^ B-CLL cells and p53^mutated/null^ leukemic cell lines, at levels comparable to primary p53^wild-type^ B-CLL cells, in a range of concentrations previously used by other authors in *in vitro* models of solid tumors [10-12,14]. These findings are particularly remarkable since patients harboring TP53 mutations have a poor prognosis and still limited therapeutic options [18]. In addition, we have demonstrated that DCA induces cell cycle arrest in the G1 phase, senescence, apoptosis, also independently of p53. Moreover, although it has been previously hypothesized that the lack of mitochondrial hyperpolarization in hematological malignancies might render DCA ineffective in such cases [26], DCA efficiently induced mitochondrial damage in all p53^mutated/null^ leukemic cell lines investigated. Indeed, we documented both the morphological remodeling of mitochondrial structure and the reduction in ATP synthesis. Consistently, also the proteomic analyses revealed a relevant modulation of proteins involved in the metabolic processes including proteins of the mitochondrial inner membrane, such as adenine nucleotide translocase 2 (ANT2) [27] and nicotinamide nucleotide transhydrogenase (NNT) [28]. Overall, these data support the notion that DCA cytotoxity in p53^mutated^ B-CLL cells is also to be ascribed to the metabolic activity of this compound.

Even though we have previously demonstrated that DCA activated the p53 pathway and promoted the transcription of several p53 target genes in p53^wild-type^ B leukemic cells [15], we have here demonstrated that DCA induced the transcription of p21 also in a p53-independent manner in both primary p53^mutated^ B-CLL patient cells and p53^mutated/null^ leukemic cell lines. Another novel finding of our study was the identification of the transcription factor ILF3 as a key protein regulating p21 expression in leukemic cells and in response to exposure to DCA. Indeed, the knock-down of ILF3 in p53^null^ HL-60 cells resulted in the attenuation of p21 expression as well as in the significant decrease of DCA-mediated cytotoxicity.

Recent studies confirmed that p21 is necessary for the induction of mitochondrial dysfunction observed in senescence and this event maintains a constant DNA damage response leading to prolonged cell cycle arrest [29]. P21 prevents cancer cell growth due to its ability to transiently or permanently stop proliferation, thus being an important component of tumor suppressor mechanisms. Indeed, it has recently been demonstrated that p21 can be down-regulated by several microRNA expressed in cancer cells [30,31]. Concurrently, although p21 levels have been found elevated in some cancers without signs of growth inhibition [32] and in some experimental settings it has been proposed that p21 can actually favor transformation by inhibiting apoptosis [31], other studies have recently shown that p21 can also trigger cell death [20,29]. At present, it is not well understood how p21 exerts these radically different functions or even if they reside in separate domains of the protein.

In spite of the discrepancies present in the scientific literature, our current data clearly point to the tumor-suppressor activity of p21 in the context of DCA-induced cytotoxicity on p53^mutated^ primary B-CLL cells as well as on p53^mutated/null^ leukemic cell lines. In agreement with our current data, it has been demonstrated that the *in vitro* activation of p21 in B-CLL accurately predicts the therapeutic response *in vivo* of B-CLL [20]. Since p53 mutations still represent one of the major negative prognosticators of B-CLL, we believe that the therapeutic potential of DCA should be further evaluated possibly in combination with other innovative anti-leukemic drugs.

## METHODS

### Primary B-CLL patient samples and leukemic cell lines

For experiments with primary cells, peripheral blood samples were collected in heparin-coated tubes from B-CLL patients (n=10) following informed consent, in accordance with the Declaration of Helsinki and in agreement with institutional guidelines (University-Hospital of Ferrara). The patients had been without prior therapy at least for three weeks before blood collection. Peripheral blood mononuclear cells (PBMC) were isolated by gradient centrifugation with lymphocyte cell separation medium (Cedarlane Laboratories, Hornby, ON, CAN). T lymphocytes, NK lymphocytes, granulocytes and monocytes were negatively depleted from peripheral blood B-CLL with immunomagnetic microbeads (MACS microbeads, Miltenyi Biotech, Auburn, CA), with a purity >95% of resulting CD19^+^ population, as assessed by flow cytometry analysis and previously described [33].

To analyze the TP53 status, B-CLL patients' DNA samples obtained from circulating CD19^+^ cells were sequenced on Ion Torrent Personal Genome Machine system using a custom Ion AmpliSeq panel (Life Technologies, Carlsbad, CA) targeting the exonic regions and the exon-intron boundaries of TP53. Next generation sequencing data was annotated by a standard ANNOVAR pipeline supplied by COSMIC v68 database. All selected variants were validated by Sanger sequencing, first on genomic DNA and in case of splicing variants on cDNA. The potential pathogenicity of the identified mutations and their effects on p53 functionality was predicted by web tools (SIFT, http://sift.jcvi.org/; PolyPhen-2, http://genetics.bwh.harvard.edu/pph2/; MutationTaster, http://www.mutationtaster.org/; HSF, http://www.umd.be/HSF; NNSplice, http://www.fruitfly.org/seq_tools/splice.html) and by protein structural bioinformatics analysis (NCBI, http://www.ncbi.nlm.nih.gov/protein/; PDB, http://pdb.org; UniProt, http://www.uniprot.org; PFAM, http://pfam.sanger.ac.uk). The main clinical and molecular parameters of the B-CLL patients are reported in Table 1.

For the *in vitro* assays, B-CLL patients' cells were cultured in RPMI-1640 medium containing 10% FBS, L-glutamine and Penicillin/streptomycin (all from Gibco, Grand Island, NY) and used within the first 48 hours of cultures when the mean (±SD) percentage of cell viability was between 96.5% (±1.8%, in the first 24 hours) and 82.5% (±7.3%, at 48 hours).

The p53^mutated^ B lymphoblastoid leukemic cell lines MAVER, MEC-1 and MEC-2 and the p53^null^ HL-60 leukemic cell line were purchased from DSMZ (Deutsche Sammlung von Mikroorganismen und Zellkulturen GmbH, Braunschweig, Germany). MAVER and HL-60 cell lines were routinely cultured in RPMI-1640, whereas MEC-1 and MEC-2 were maintained in IMDM, all supplemented with 10% FBS, L-glutamine and Penicillin/streptomycin (all from Gibco). Cells were then treated with DCA (range 1-30 mM; Sigma-Aldrich, St Louis, MO), and, for selected experiments, with Nutlin-3 (10 μM; Cayman Chemicals, Ann Arbor, MI).

### Analysis of DCA cytotoxicity

For the *in vitro* assessment of cytotoxicity, at different time points post DCA treatment, overall cell viability was examined by Trypan blue dye exclusion and MTT assay as previously described [34]. Apoptosis and cell cycle were analyzed in flow cytometry by Annexin V-FITC/propidium iodide (PI) staining (Immunotech, Marseille, France) and 5-bromodeoxyuridine (BrdU; Sigma-Aldrich) incorporation, respectively [35,36], while senescence was assessed under light microscope by senescence-associated β-galactosidase (SA-β-gal) staining, using a specific kit (Abcam, Cambridge, UK) and following the manufacturer's instructions.

### Assessment of mitochondrial alterations

The presence of morphological signs characteristic of mitochondrial alteration, such as mitochondrial fragmentation and cristae remodeling, were analyzed by transmission electron microscopy [37]. Mitochondrial activity was evaluated by measuring the cellular ATP production. Briefly, ATP concentration was determined using the ATP Assay KIT (Abcam) in cell lysates of untreated and DCA treated cultures at different time points. Measurements were performed with the colorimetric method, following the manufacturer's instructions. ATP contents were normalized per number of cells.

### RT-PCR and Western blot analyses

Total RNA was extracted from cells using the QIAGEN RNeasy Plus mini kit (QIAGEN, Hilden, Germany). For RT-PCR, total RNA was transcribed into cDNA, using the QuantiTect^®^ Reverse Transcription kit (SABiosciences, QIAGEN). P21 and ILF3 gene expression was analyzed using the SYBR Green-based RT qPCR detection method with SABiosciences RT^2^ Real-Time^TM^ Gene expression assays, which include specific validated primer sets and PCR master mix (SABiosciences). All samples were run in triplicate using the real-time thermal analyzer Rotor-Gene^TM^ 6000 (Corbett, Cambridge, UK), as previously described [37]. Expression values were normalized to the housekeeping gene POLR2A amplified in the same sample.

For Western blot analysis, cells were lysed as previously described [38]. Protein determination was performed by using the BCA Protein Assay (Thermo Scientific, Rockford, IL). Equal amounts of protein for each sample were migrated in SDS-polyacrylamide gels and blotted onto nitrocellulose filters, as previously described [39]. The following Abs were used: anti-p21 (C-19) and anti-tubulin purchased from Santa Cruz Biotechnology (Santa Cruz, CA) and from Sigma-Aldrich, respectively. After incubation with anti-mouse IgG horseradish peroxidase-conjugated secondary Abs (Sigma-Aldrich), specific reactions were revealed with the ECL Lightning detection kit (Perkin Elmer, Waltham, MA) [40]. Densitometry values were estimated by the ImageQuant TL software (GE Healthcare, Buckinghamshire, UK).

### Proteomic profiling

To perform one-dimensional gel electrophoresis (1-DE), for each experimental group triplicates of HL-60 cell protein extract (25 μg/replicate) were mixed with equal volume of Laemmli sample buffer and resolved on a NuPAGE® Novex® 4-12% Bis-Tris gel (Life Technologies). Each gel lane was manually cut with a sterile surgical blade into 24 bands of equal height (about 3 mm) and excised bands were crushed into small fragments, processed, submitted to in-gel trypsin digestion and peptide extractions. An aliquot of each digest (2 μl) was directly analyzed by liquid chromatography-tandem mass spectrometry (LC-MS/MS) using a LTQ Orbitrap XL™ (Thermo Scientific), interfaced with a 1200 series capillary pump (Agilent Technologies Inc., Santa Clara, CA).

For protein identification, tandem mass spectra were extracted and the charge states deconvoluted by BioWorks version 3.3.1 (Thermo Scientific). For each sample, the MS/MS data from the 24 gel bands were merged and submitted as “mgf” file to the search engine Mascot (in-house version 2.2.06, Matrix Science, London, UK). Scaffold (version 3_00_02, Proteome Software Inc., Portland, ME) was used to validate MS/MS-based peptide and protein identifications. Mascot was set up to search both the forward and reversed sequences of the SwissProt_57.8 database (selected for Humans) assuming the digestion enzyme trypsin (1 missed cleavage allowed), mass tolerance of 1.00 Da and a parent ion tolerance of 2.0 ppm. Carbamidomethylation of cysteine was specified as a fixed modification and deamidation of asparagine and oxidation of methionine were set as variable modifications in both search engines. Peptide identifications were accepted if they could be established at greater than 95.0%. Protein identifications were accepted if established at greater than 99.9% probability with at least 2 identified peptides. Identified proteins were quantified using spectral counts directly computed by Scaffold and the number of MS/MS spectra were grouped into “counts” associated to a specific protein. Spectral counts normalization was performed by Scaffold 8 version 3_00_02 (Proteome Software Inc.). Subsequently estimation of differential protein abundance was expressed as fold-change (ratio of the averaged spectral counts in the treated cells to the averaged spectral counts in the untreated cells) (Supplementary Table 1). The normalized spectral count for each identified protein were submitted to SIMCA-P13 software package (Umetrics, Umea, Sweden) for multivariate data analysis (OPLS-DA). Variables that had significant contribution to discrimination between groups and a significant p-value (Mann-Whitney-Wilcoxon test, p<0.05), computed using JMP v6 software (SAS Institute, Inc., Cary, NC), were accepted as significantly modulated proteins upon treatment and submitted to network analysis (Supplementary Table 2). Functional interpretation of the differentially expressed proteins highlighted by proteomics was built using the MetaCore analytical suite version 5.3 (GeneGo, St. Joseph, MI).

### Transfection experiments

A pool of three specific small interfering RNA (siRNA) for each gene was resuspended in nuclease-free sterile water. Leukemic cells (2×10^6^ cells/0.1 ml) were mixed with either 1 μg of control enhanced green fluorescence protein (EGFP) plasmid or 3 μg of siRNA, transferred to a 2.0-mm electroporation cuvette and nucleofected with the nucleofector kit V, using the nucleofector device (Amaxa Nucleofector II apparatus, Lonza Cologne AG, Cologne, Germany), as previously described [41]. Transfection efficiency was monitored by scoring the percentage of EGFP-positive cells by flow cytometry analysis as previously described [42]. For p21 and ILF3 gene knock-down, siRNAs were designed and manufactured by Ambion^®^ (Life Technologies) according to the guidelines for effective gene knock-down by this method. The silencer negative control siRNA consisted of a 19 bp-scrambled sequence with 3′ dT overhangs and previously tested for the lack of non-specific effects on gene expression.

### Statistical analysis

Descriptive statistics were calculated. For each set of experiments (ie, assays for cell cytotoxicity/functionality and RT-analysis), values were reported as means±standard deviation (SD). The results were analyzed by using the Mann-Whitney rank-sum test and statistical significance was defined as p<0.05. All statistical analyses were performed with SPSS Statistic 20 software (SPSS Inc., Chicago, IL). Proteomics data were analyzed by both multivariate and univariate approaches. The normalized spectral counts for each identified proteins were submitted to SIMCA-P13 software package (Umetrics, Umea, Sweden) for multivariate data analysis. Variables were scaled using Pareto scaling and data were analyzed by orthogonal partial least-squares discriminant analysis (OPLS-DA). S-plots were calculated to visualize the relationship between covariance and correlation within the OPLS-DA results. Variables that showed significant contribution to discrimination between groups and a significant change in their expression (Mann-Whitney-Wilcoxon test, p<0.05), were accepted as significantly modulated proteins upon treatment and submitted to network analysis.

## SUPPLEMENTARYMATERIAL, TABLES AND FIGURES


