# Supplementary material for: The anti-leukemic activity of sodium dichloroacetate in p53mutated/null cells is mediated by a p53-independent ILF3/p21 pathway
Source: Oncotarget. 2014 Dec 10;6(4):2385–96. doi: 10.18632/oncotarget.2960 (PMC4385858; doi:10.18632/oncotarget.2960)
Supplement: Supplementary file 1 [file oncotarget-06-2385-s001.pdf]

The anti-leukemic activity of sodium dichloroacetate in p53[mutated/null] cells is mediated by a p53-independent ILF3/p21 pathway

Supplementary Material

Supplementary Table 1: Differential expression data (as normalized spectral counts) for each identified protein whose abundance was significantly altered in HL-60 cells by DCA treatment

Protein name	UniprotKB	HL-60 Untreated						HL-60 DCA30mM						Fold regulation[g]
		1R[a] NSC[d]	2R[b] NSC[d]	3R[c] NSC[d]	Average NSC	SD[e]	%CV[f]	1R[a] NSC[d]	2R[b] NSC[d]	3R[c] NSC[d]	Average NSC	SD[e]	%CV[f]	
ADP/ATP translocase 2 (ANT2)	P05141	26	35	37	32.67	5.86	17.94	1	1	1	1.00	0.00	0.00	-32.67
ADP-ribosylation factor 4	P18085	1	1	1	1.00	0.00	0.00	6	6	5	5.67	0.58	10.19	5.67
ADP-ribosylation factor 5	P84085	1	1	1	1.00	0.00	0.00	8	8	6	7.33	1.15	15.75	7.33
Asparagine-tRNA ligase, cytoplasmic	O43776	4	4	5	4.33	0.58	13.32	17	20	21	19.33	2.08	10.77	4.46
Endoplasmic reticulum resident protein 44	Q9BS26	9	7	7	7.67	1.15	15.06	2	1	3	2.00	1.00	50.00	-3.83
Eukaryotic translation initiation factor 2 subunit 3	P41091	3	2	2	2.33	0.58	24.74	5	9	9	7.67	2.31	30.12	3.29
Glycine-tRNA ligase	P41250	1	1	1	1.00	0.00	0.00	9	10	11	10.00	1.00	10.00	10.00
Growth factor receptor-bound protein 2	P62993	7	6	7	6.67	0.58	8.66	1	1	1	1.00	0.00	0.00	-6.67
Histidine--tRNA ligase, cytoplasmic	P12081	5	6	8	6.33	1.53	24.12	1	1	1	1.00	0.00	0.00	-6.33
Histone H3.1	P68431	1	1	1	1.00	0.00	0.00	8	7	4	6.33	2.08	32.87	6.33
Histone H3.2	Q71DI3	1	1	1	1.00	0.00	0.00	8	5	5	6.00	1.73	28.87	6.00
Interleukin enhancer-binding factor 3	Q12906	1	3	1	1.67	1.15	69.28	11	7	5	7.67	3.06	39.85	4.60
NAD(P) transhydrogenase, mitochondrial (NNT)	Q13423	1	1	1	1.00	0.00	0.00	5	9	8	7.33	2.08	28.39	7.33
Prothymosin alpha	P06454	1	1	1	1.00	0.00	0.00	8	17	17	14.00	5.20	37.12	14.00
Ribonucleoside-diphosphate reductase large subunit	P23921	9	13	11	11.00	2.00	18.18	4	4	3	3.67	0.58	15.75	-3.00
Serine/threonine-protein phosphatase PP1-alpha catalytic subunit	P62136	14	12	12	12.67	1.15	9.12	9	1	1	3.67	4.62	125.97	-3.45
Tubulin-tyrosine ligase-like protein 12	Q14166	9	10	8	9.00	1.00	11.11	2	4	3	3.00	1.00	33.33	-3.00

[a],first replicate; [b],second replicate; [c],third replicate; [d],number of normalized spectral counts (NSC); [e],standard deviation of the averaged NSC; [f],coefficient of variation (CV%); [g],fold regulation in expression HL60 DCA treated cells vs non-treated cells.

Supplementary Table 2. Proteins significantly altered (OPLS-DA, Mann-Whitney, $p<0,05$) in HL-60 cells after DCA treatment

Description	Accession Number	UniProtkB	Fold regulation
ADP/ATP translocase 2 (ANT2)	ADT2_HUMAN	P05141	-32.67
ADP-ribosylation factor 4	ARF4_HUMAN	P18085	5.67
ADP-ribosylation factor 5	ARF5_HUMAN	P84085	7.33
Asparagine-tRNA ligase, cytoplasmic	SYNC_HUMAN	O43776	4.46
Endoplasmic reticulum resident protein 44	ERP44_HUMAN	Q9BS26	-3.83
Eukaryotic translation initiation factor 2 subunit 3	IF2G_HUMAN	P41091	3.29
Glycine-tRNA ligase	SYG_HUMAN	P41250	10.00
Growth factor receptor-bound protein 2	GRB2_HUMAN	P62993	-6.67
Histidine-tRNA ligase, cytoplasmic	SYHC_HUMAN	P12081	-6.33
Histone H3.1	H31_HUMAN	P68431	6.33
Histone H3.2	H32_HUMAN	Q71DI3	6.00
Interleukin enhancer-binding factor 3	ILF3_HUMAN	Q12906	4.60
NAD(P) transhydrogenase, mitochondrial (NNT)	NNTM_HUMAN	Q13423	7.33
Prothymosin alpha	PTMA_HUMAN	P06454	14.00
Ribonucleoside-diphosphate reductase large subunit	RIR1_HUMAN	P23921	-3.00
Serine/threonine-protein phosphatase PP1-alpha catalytic subunit	PP1A_HUMAN	P62136	-3.45
Tubulin-tyrosine ligase-like protein 12	TTL12_HUMAN	Q14166	-3.00

Data report proteins differentially modulated (above the cutoff of 3 fold of induction or below the cutoff of -3 fold of reduction) after DCA 30mM treatment in HL-60 cells.

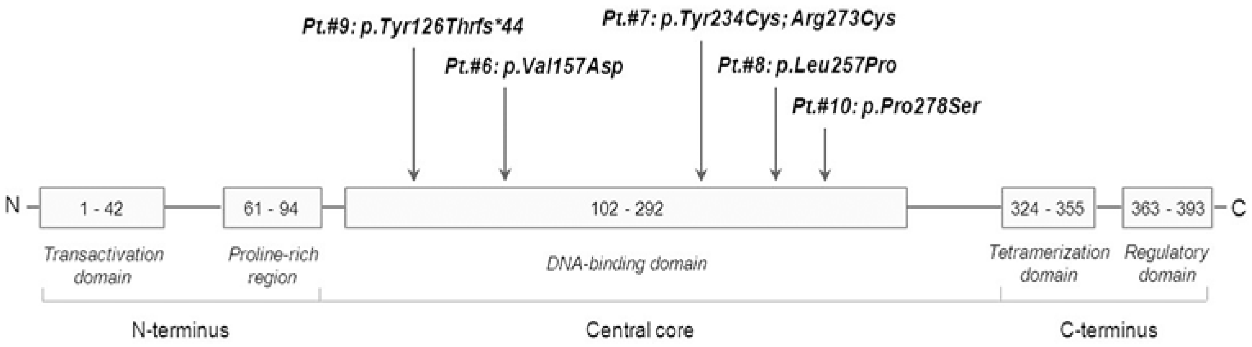

Supplementary Figure 1: Sites of mutation in p53 in the B-CLL patients investigated. A schematic representation of the 393 amino acid domain structure of human p53 is shown. The sites of the mutations detected in the B-CLL patients (Pt.#6-10) are indicated. The nomenclature follows the current HGVS recommendations for the description of sequence variants.

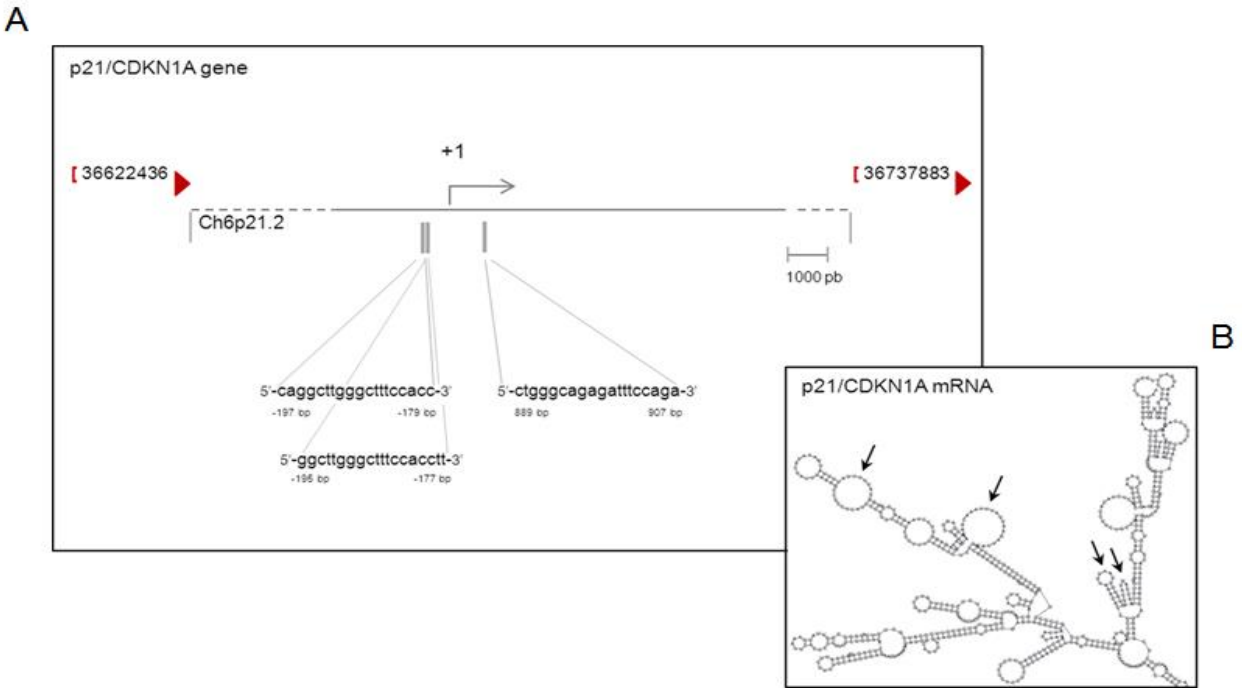

Supplementary Figure 2: Mechanisms of p21/CDKN1A gene regulation by ILF3. Schematic representation of the predicted ILF3 binding sites on the regulatory region of the human p21/CDKN1A gene (A) and on the p21 mRNA 3’-UTR secondary structure (B), supporting the transcriptional and post-transcriptional regulation of p21/CDKN1A by ILF3. In A, the purine-rich NF-AT/ILF3 binding sites, predicted by computational analysis (MatInspector analyses of Genomatix), are evidenced in the regulatory region of the human p21/CDKN1A gene. In B, mRNA 3’-UTR secondary structure analysis (RNA fold program from the Vienna RNA Package) of a partial region of p21/CDKN1A is represented; arrows indicate the AU-rich elements in the sequence recognized by ILF3, involved in the stabilization of p21 mRNA.

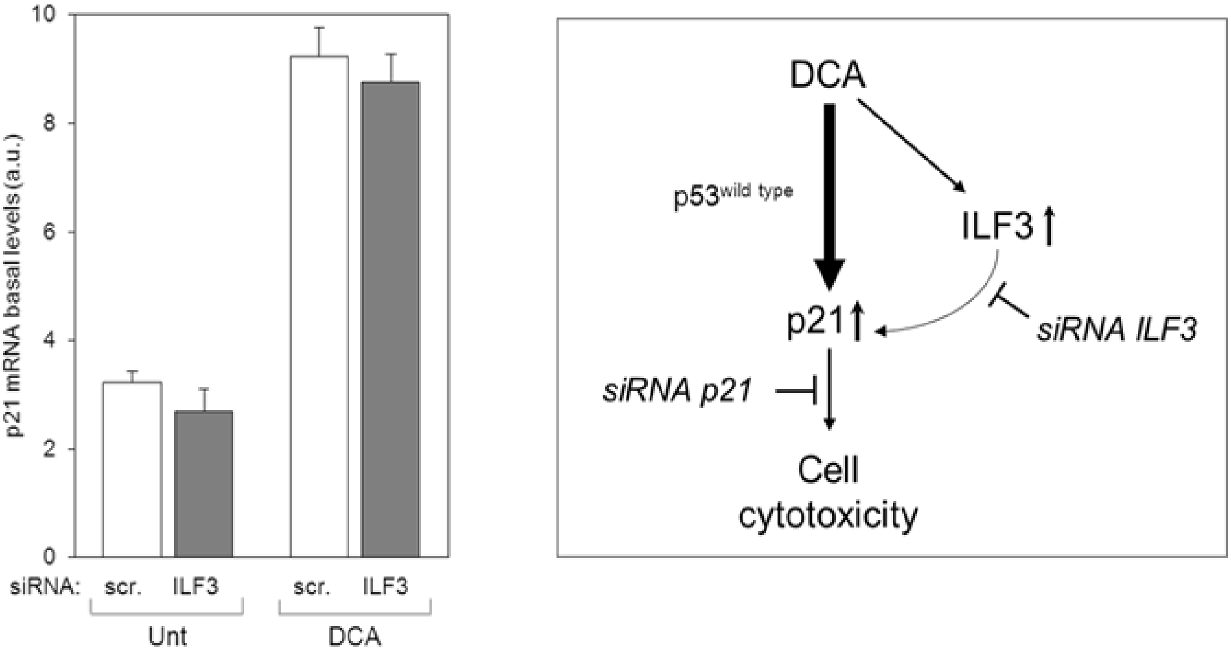

Supplementary Figure 3: Analysis of the DCA-ILF3-p21 axis in $p53^{wild-type}$ leukemic cells. In A, $p53^{wild-type}$ JVM-2 cells were transfected with either control scrambled (scr) siRNA or ILF3 siRNA , before treatment with DCA (30 mM). Upon ILF3 silencing, levels of p21 mRNA were assessed by quantitative RT-PCR, both in untreated and DCA treated cells. Data are expressed as arbitrary units (a.u.) and reported as means±SD of results from three independent experiments, each performed in duplicate. In B, a schematic representation of the hypothetic p53-dependent and p53-independent pathways mediating the p21 modulation in response to DCA.
